# Geographical and temporal patterns of rabies post exposure prophylaxis (PEP) incidence in humans in the Mekong River Delta and Southeast Central Coast regions in Vietnam from 2005 to 2015

**DOI:** 10.1371/journal.pone.0194943

**Published:** 2018-04-10

**Authors:** Hu Suk Lee, Vu Dinh Thiem, Dang Duc Anh, Tran Nhu Duong, Mihye Lee, Delia Grace, Hung Nguyen-Viet

**Affiliations:** 1 International Livestock Research Institute, Hanoi, Vietnam; 2 National Institute of Hygiene and Epidemiology, Hanoi, Vietnam; 3 Medical Microbiology Department, The Royal Bournemouth Hospital, Bournemouth, United Kingdom; 4 International Livestock Research Institute, Nairobi, Kenya; Wistar Institute, UNITED STATES

## Abstract

**Background:**

In Vietnam, rabies has been a notifiable disease for more than 40 years. Over the last five years, on average, more than 350,000 people per year have been bitten by dogs and cats while more than 80 human deaths have been reported yearly. No studies have been conducted to evaluate the geographical and temporal patterns of rabies in humans in Vietnam. Therefore, the main objective of this study was to assess the geographical and temporal distributions of rabies post exposure prophylaxis (PEP) incidence in humans in Vietnam from 2005 to 2015.

**Methods:**

Average incidence rabies (AIR) PEP rates for every 3 or 4 years (2005–2008, 2009–2012 and 2013–2015) were calculated to describe the spatial distribution of rabies PEP. Hotspot analysis was implemented to identify patterns of spatial significance using the Getis-Ord Gi statistic. For temporal pattern analysis, two regions [Mekong River Delta (MRD) and Southeast Central Coast (SCC)], with the highest incidence rates, and the seasonal-decomposition procedure based on loess (STL), were compared to assess their temporal patterns of rabies PEP.

**Findings:**

We found hotspots in southern Vietnam and coldspots in northern Vietnam during the study period. Rabies cases were limited to specific areas. In addition, the hotspot analysis showed that new risk areas were identified in each period which were not observed in incidence rate maps. The seasonal plots showed seasonal patterns with a strong peak in February/July and a minor peak in October/December in the MRD region. However, in the SCC, a small peak was detected at the early part of each year and a strong peak in the middle of each year.

**Conclusion:**

Our findings provide insight into understanding the geographical and seasonal patterns of rabies PEP in Vietnam. This study provides evidence to aid policy makers when making decisions and investing resources. Such information may also be utilized to raise public awareness to prevent rabies exposures and reduce unnecessary PEP.

## Introduction

Rabies is a neglected viral zoonosis with an estimated annual 59,000 human deaths (95% confidence intervals: 25–159,000) [1]. Over 99% of rabies deaths occur in Africa and Asia [2, 3]. Effective and economical preventive measures are available, but rabies remains a major public health concern in most developing countries [4–8]. Two major epidemiological forms have been identified: an urban cycle involving dogs; and a sylvatic cycle involving wildlife [9]. Bites of infected dogs are the main source of human rabies, causing up to 95% of cases [4, 10, 11].

In Vietnam, rabies is endemic and has been a notifiable disease for more than 40 years. One molecular study suggested that rabies virus (RV) in Vietnam and Thailand are closely related [12]. The key factors contributing to the disease include a low vaccination coverage in dogs, a lack of public awareness and access to human rabies immune globulin (RIG) and vaccine [4, 13]. According to a national report, ver the last five years, more than 350,000 people per year have been bitten by dogs and cats, while more than 80 human deaths have been reported annually. According to the Department of Animal Health, only 29% (2.9 million) of dogs have been vaccinated of an estimated population of 10 million dogs. Currently, Vietnam is involved in the Association of South East Asian Nations (ASEAN) action plan for rabies elimination by 2020 [14]. The Ministry of Health (MOH) and Ministry of Agriculture and Rural Development (MARD) organized a national five year strategy of rabies elimination for 2017–2021 using the Stepwise Approach to Rabies Elimination (SARE), a key initiative to understand the complexities of rabies control and to develop a platform that makes rabies control programs more effective and manageable [15].

In Vietnam, relatively few studies on rabies have been published. Most were case reports or related to evaluation of public awareness and molecular epidemiology of rabies in humans and dogs in Vietnam [12, 16–18]. To our knowledge, no studies have been conducted to evaluate the geographical and temporal patterns of rabies in humans in Vietnam. Therefore, the main objective of this study was to assess the geographical and temporal distributions of rabies post exposure prophylaxis (PEP) in human in the regions where the highest incidence rates were recorded from 2005 to 2015 in Vietnam. These data should help in the planning and targeting of rabies prevention and control efforts.

## Materials and methods

### Study location and data

Vietnam is located on the eastern Indochina Peninsula and is a long, narrow nation with an estimated population of 91.7 million in 2015 [19]. Monthly cases and incidence rates for rabies at a provincial level from 2005 to 2015 were obtained from the Vietnam annual infectious diseases books, published by the MOH. Human rabies is a notifiable disease (one of 28 notifiable diseases in Vietnam), which is reported on a weekly basis by the preventive medicine center networks. According to the World Health Organization (WHO), the rabies case definition is a patient presenting with an acute neurological syndrome (encephalitis) or paralytic syndrome progressing towards coma and death, usually by respiratory failure, within 7–10 days after the first symptom [4]. In Vietnam, rabies PEP cases are recorded on the basis of a consultation with a doctor and resultant administration of PEP within 3 days after an animal bite (mainly dogs). Most cases are not laboratory confirmed. The numbers of PEP and death cases are collected by provincial preventive medicine centers which report to the regional preventive medicine institutes, and then to the National Institute of Hygiene and Epidemiology (NIHE). The MOH annually publishes reported rabies PEP cases and incidence rates (per 100,000). These data were entered into an Excel spreadsheet by our researchers.

### Data analysis

The eleven–year period was divided into three-year periods to efficiently present data. Therefore, average incidence rabies (AIR) PEP rates for every 3 or 4 years (2005–2008, 2009–2012 and 2013–2015) were calculated to describe the spatial distribution of rabies PEP. For this analysis, the population at provincial levels was reversely extrapolated from PEP cases and incidence rates (per 100,000) as monthly cases and incidence rates were only recorded in the books. These were used to estimate the average incidence rates for every 3 or 4 year period. The geographical distribution of rabies PEP at a provincial level was graphically represented using ArcGIS software. Hotspot analysis was implemented to identify patterns of spatial significance at a provincial level using the Getis-Ord Gi statistic in ArcGIS software [20]. This statistic makes a comparison between a local mean rate (the rates for a province and its nearest neighboring provinces) and a global mean rate (the rates for all provinces). A hotspot/coldspot was defined as an area that has a higher/lower concentration of events compared to the expected number given a random distribution of events.

**Fig 1:**
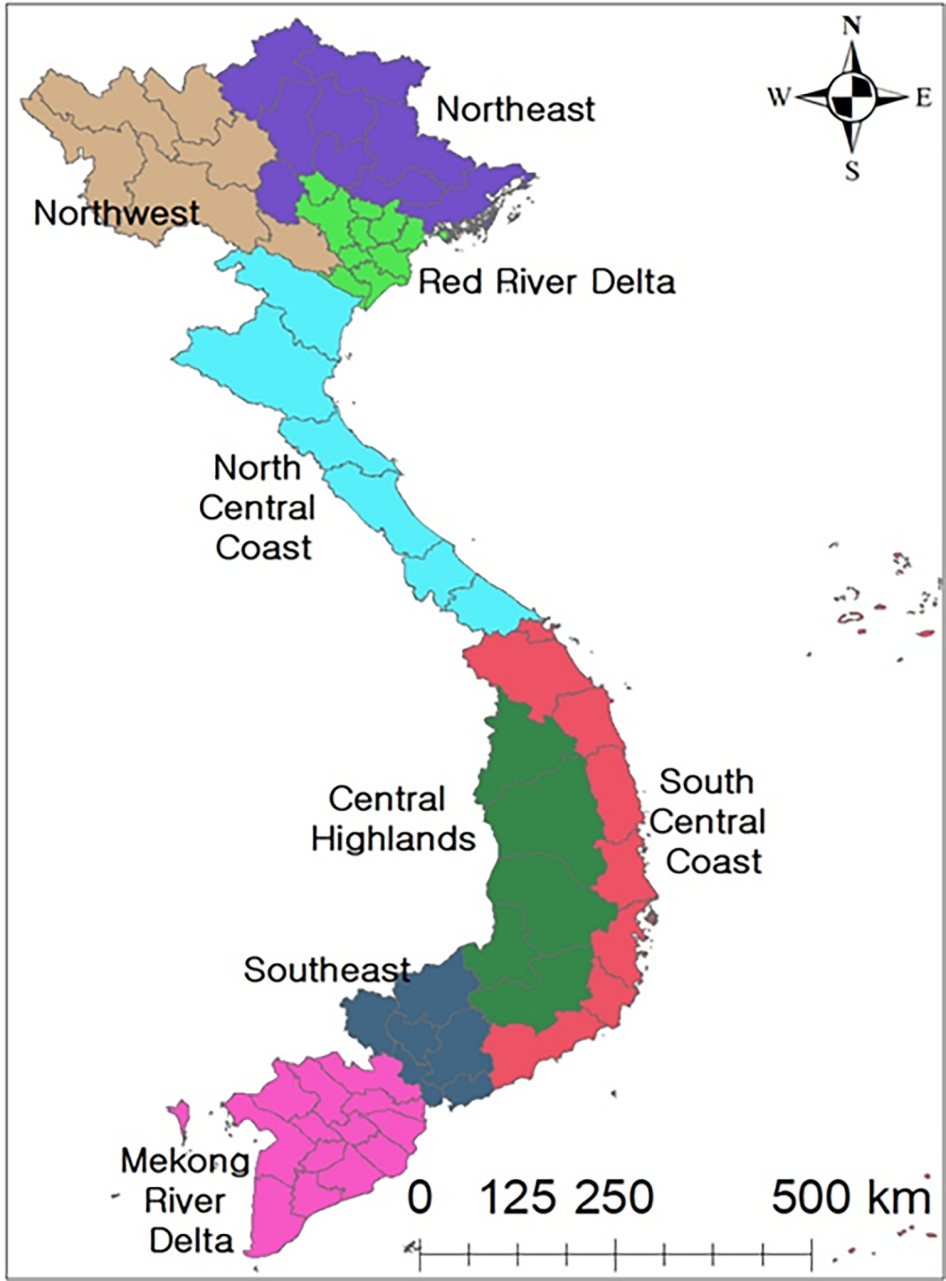
Lists of regions in Vietnam.

For the temporal pattern analysis, Vietnam is divided into 63 provinces and these provinces are allocated to 8 eco-regions based on the similarities of geographical features and climate conditions [21] (Fig 1). We selected regions with relatively high PEP incidences. The seasonal-decomposition procedure based on loess (STL) was implemented by each region to assess the temporal patterns of rabies PEP. The STL method is used to decompose time series data into trend, seasonal and remainder on a yearly basis (12 months) [22]. A seasonal cycle subseries plot (SCS) was used to evaluate the monthly variations by each region. In addition, statistical analysis (using a univariate negative binomial regression (NBR) model) was employed to assess the monthly differences [23]. Incidence rate ratio (IRR) and 95% confidence intervals (CI) were calculated by exponentiation of the regression coefficients. January was used as a reference due to a relatively low rate. If the p-value was less than 0.05, it was considered to be significant.

All data were imported into Microsoft Excel 2013 and were analyzed using R (version 3.2.2) and STATA (version 14.2, StataCorp, College Station, TX, USA). ArcGIS version 10.4.1 ArcMap (ESRI, Redlands, CA, USA) was used to generate maps as well as to conduct hotspot analysis. The study was approved by the Hanoi Medical University Institutional Review Board (reference number: 00003121; approval date: 22 Dec 2015), Vietnam.

## Results

### Geographic distribution of rabies PEP incidence

A total of 4,196,247 (average 429.55 per 100,000) PEP cases and 934 deaths (average 0.10 per 100,000) were reported from 2005 to 2015. Overall, PEP incidence rates have decreased continuously over time from 654.92 to 389.37 per 100,000, while death rates showed less fluctuations from 0.09 to 0.08 per 100,000. Ben Tre province (average: 1562.72 per 100,000) showed the highest PEP incidence rate followed by Binh Thuan (average: 1513.07 per 100,000), whereas Thai Binh (average: 22.05 per 100,000) and Kon Tum (average: 39.82 per 100,000) had the lowest PEP incidence rates during the study period.

**Fig 2:**
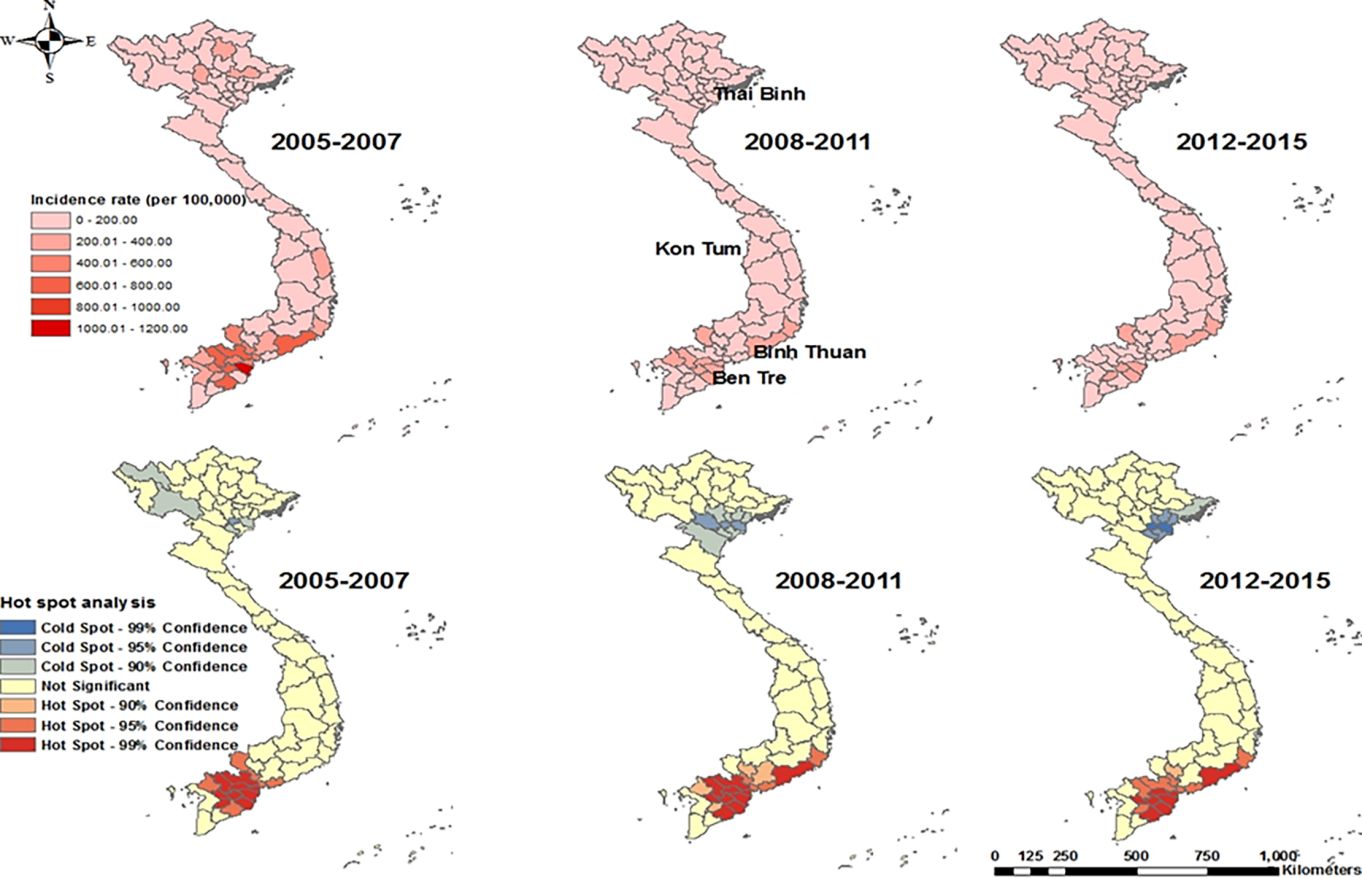
Average rabies PEP incidence rates (per 100,000) (upper maps) and hotspot analysis (lower maps) of rabies PEP in Vietnam, from 2005.

The MRD and SCC regions showed relatively high incidence rates, whereas some north and central regions had relatively low incidence rates (Fig 2: upper map). According to the hotspot analysis (Getis-Ord Gi statistic), a total of 16 provinces from the Mekong River Delta (MRD) and Southeast Central Coast (SCC) regions were identified as PEP hotspots, whereas one province from the North region was classified as a PEP coldspot between 2005 and 2007 (Fig 2: lower map) at a 5% level. Thirteen provinces from the same region were classified as hotspots, whereas 3 provinces from the north region were identified as PEP coldspots from 2008 to 2011. Lastly, 14 provinces from the same region were classified as PEP hotspots, whereas 5 provinces from the north region were classified as PEP coldspots during the 2012–2015 period.

### Temporal patterns in the Mekong River Delta and Southeast Central Coast regions

**Fig 3:**
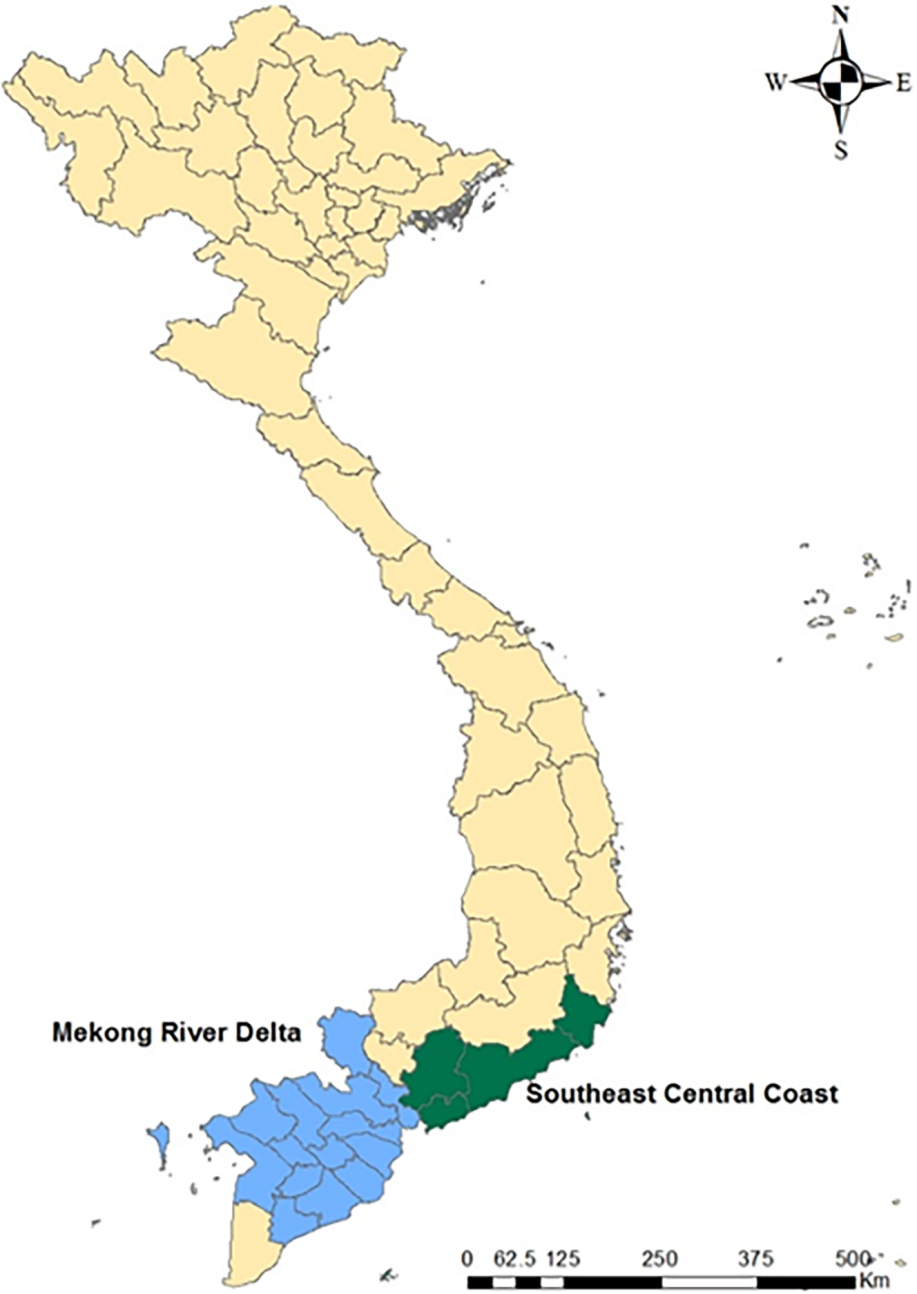
Highest rabies PEP incidence areas in Mekong River Delta and Southeast Central Coast.

**Fig 4:**
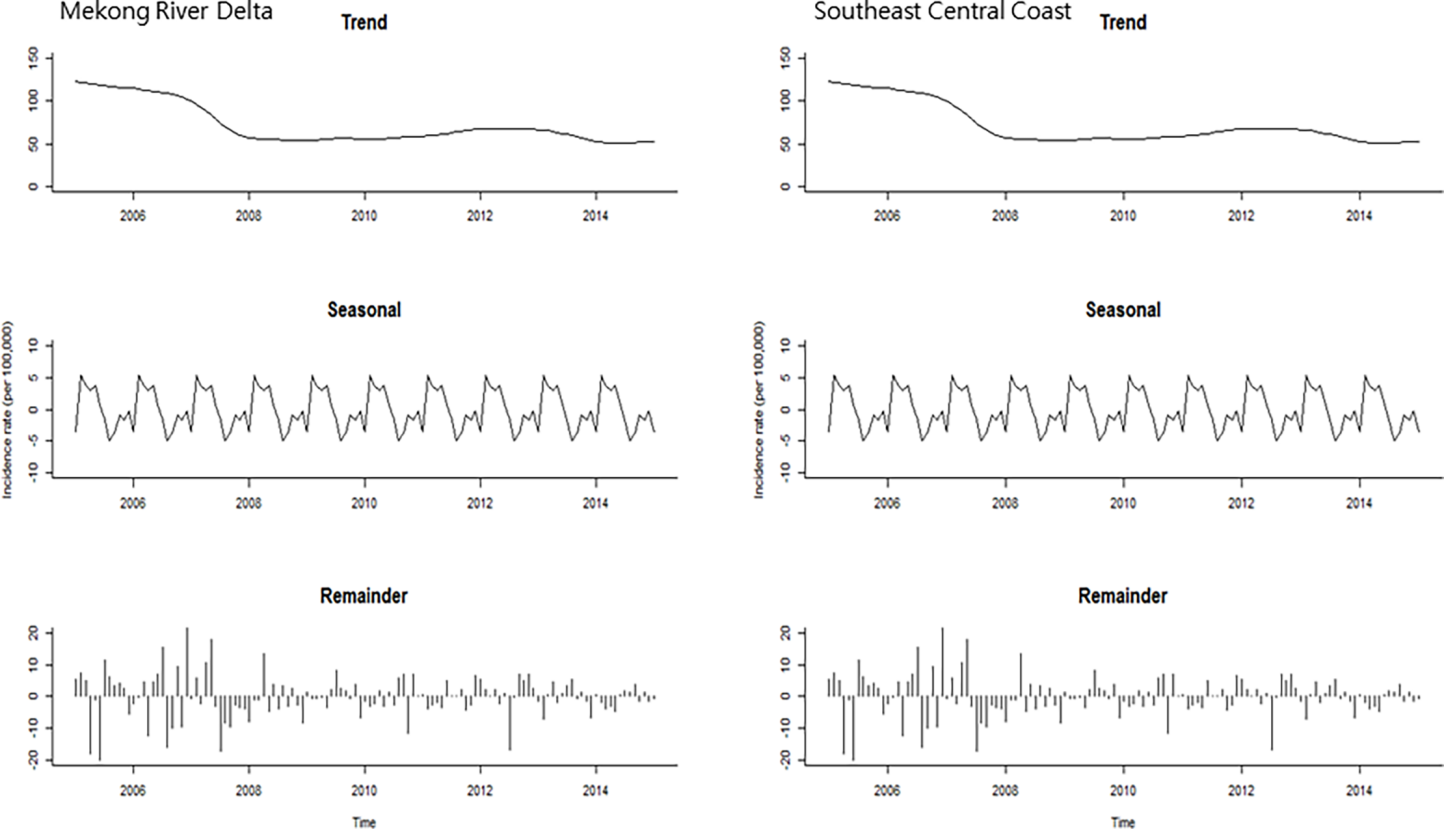
Seasonal-trend decomposition of the monthly incidence rates (per 100 000) of rabies PEP, 2005–2015.

**Fig 5:**
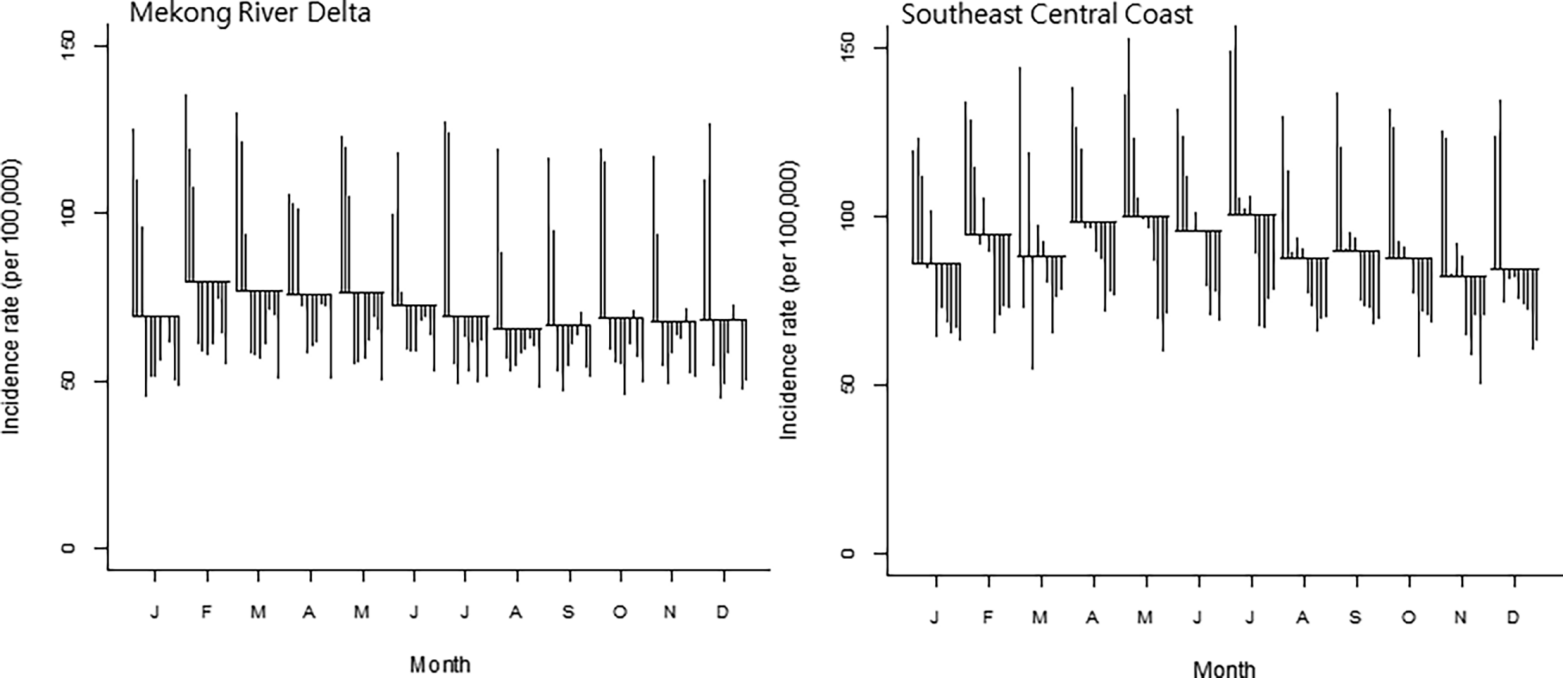
Seasonal cycle subseries plot of the monthly incidence rates (per 100 000) of rabies PEP, 2005–2015 (vertical line: incidence rate for the same month in each year; horizontal lines: Average for vertial lines).

For seasonality analysis, provincial level data were merged into two regions (MRD and SCC) where the highest incidence rates were observed during the study period (Fig 3). The trend plots showed that there was a gradual decrease in both regions during the study period (Fig 4: first plot). The seasonal plots showed seasonal patterns with a strong peak in February/July and a minor peak in October/December in the MRD region (Fig 4: second plot). However, in the SCC, the small peak was detected at the early part of each year and a strong peak in the middle of each year (May-July). The remainder component showed relatively random variations in residuals, except for 2005 and 2009 with large values in the MRD region (Fig 4: third plot). For the SCC, it had random residuals with intermittently large values, especially early in 2006 and 2008 (Fig 4: third plot). The SCSs showed relatively high incidence rates between February and May compared to other months in the MRD region (Fig 5: left). In the SCC, the SCSs appeared to have relatively high incidence rates from April to July (Fig 5: right). For the univariate NRB analysis, incidence rates showed the highest from March to July in the MRD and May in the SCC (Table 1). However, none of the months were significantly different compared to January in both regions.

**Table 1:** Univariate negative binomial regression (NBR) models for the rabies PEP incidence rates by month with incidence rate ratio (IRR) and 95% confidence interval (CI).

Month	Mekong River Delta	Southeast Central Coast
January	Reference: 1	Reference: 1
February	0.98 (0.86–1.11)	1.03 (0.80–1.33)
March	1.09 (0.96–1.24)	1.10 (0.86–1.42)
April	1.09 (0.96–1.24)	1.09 (0.84–1.40)
May	1.09 (0.96–1.23)	1.19 (0.92–1.53)
June	1.09 (0.96–1.24)	1.15 (0.89–1.48)
July	1.03 (0.90–1.16)	1.12 (0.87–1.45)
August	1.01 (0.89–1.14)	1.10 (0.86–1.42)
September	0.94 (0.83–1.07)	1.01 (0.78–1.30)
October	0.92 (0.81–1.04)	1.00 (0.78–1.29)
November	0.95 (0.84–1.08)	1.02 (0.79–1.32)
December	0.99 (0.88–1.13)	0.98 (0.76–1.27)

## Discussion

This study evaluated the geographical and temporal distribution of rabies PEP in Vietnam. Overall, rabies incidence rates steadily decreased over time, probably due to increasing vaccination of dogs as well as enhanced raising public awareness. Seasonal patterns were observed in the MRD and SCC regions, with peaks in early and mid-year. Prior studies did not conduct a statistical analysis to evaluate seasonal risk differences. Our study found that there was not a significant risk difference among months. One study showed that summer and autumn had relatively high incidence which was associated with farming activities [24]. Other studies showing wildlife rabies seasonality attributed this to behaviors such as seeking food for the winter or early spring period [25–28]. During this period, due to increased movement of animals [29, 30], dogs may have had more chances to come into contact with them, which may have led to seasonal patterns of human rabies occurrence. In other countries, some studies have suggested that wildlife play a role in the transmission of human infection [31–35]. In Northern Vietnam, one study was conducted to evaluate the circulation of bat lyssaviruses. A serological study showed that a total of 193 bats (24.5%) were positive for reactivity with lyssaviruses among 789 bats [36]. Bats can serve as a reservoir, so transmission of lyssaviruses to humans is possible. Therefore, further investigation is necessary to look into the possible roles of wildlife (including bats) species in contributing to viral transmission. Other studies have suggested that the dog-breeding season (spring and summer) increased the occurrence of rabies due to fights among dogs leading to increased transmission of RV [37, 38]

We found hotspots in southern Vietnam and coldspots in northern Vietnam during the study period. It seemed that rabies cases were limited to specific areas. Our hotspot analysis showed that new risk areas were identified in each period,which were not observed in incidence rate maps. Thus, it would be worthwhile to implement a further investigation in these areas. However, it could be possible that the geographical distribution of cases was influenced by economic status, as rabies PEP cases were only recorded on the basis of clinical confirmation post-vaccination. These hotspot areas (south) were comparatively more affluent than the north while coldspots (i.e., mountainous areas) were relatively poor areas, where more ethnic minorities live. The cost for PEP is estimated to be Vietnamese Dong 1–2 million (US$ 50–100), which is a huge economic burden to people from the north (mountainous areas), whose average daily income is less than US$ 1-2/person. According to reports from the MOH, the northern mountainous region accounts for more than 80% of deaths in Vietnam, due to lack of public awareness and medical care (national data). Most cases of human rabies did not receive PEP after dog/cat bites. Therefore, this region should be targeted to efficiently reduce/eliminate rabies cases in Vietnam. Action should include providing public health education to ethnic minorities. In addition, one study found that the provincial level health workers had better knowledge/awareness compared to the district level health workers [18]. Public health workers at all levels should have ideal knowledge, attitudes and practices for the prevention, control and elimination of rabies.

Our study had several limitations. We lacked data on how many people were bitten by dogs and cats since the recorded cases were based on doctor consultations only during which PEP was administered. It is likely that bites were underestimated, especially in rural/mountain communities. A recent survey conducted in a northern central province showed that only 27% of people had received PEP after bites from dogs/cats which potentially could be rabid (not published). In addition, there was a possibility of misdiagnosis, since most of PEP cases were clinically diagnosed by doctors, with no accompanying laboratory confirmation. In the future, it is necessary to improve data collection including laboratory confirmation. In addition, we were not able to identify the distribution of age and gender of affected cases due to data limitations. According to the WHO, children (less than 15 years of age) accounted for approximately 40% of rabies cases and majority who received PEP were male [39]. It is obvious that human cases were strongly associated with the number of rabid dogs. However, we were not able to evaluate the association between human cases and dog cases/population due to a lack of basic surveillance information. We assumed that the human population was the same on a yearly basis. Although a simplification, this does not affect predictions because of the large denominators.

Our study found seasonal patterns but not significant risk differences among months in two regions during the study period. This may indicate that risk is not extremely seasonal in Vietnam or this may be an artifact of the small number of cases.

According to the WHO, high risk groups (such as veterinarians and animal handlers) should be vaccinated. The national program for rabies prevention and control in Vietnam specifies that dog slaughterhouse workers should be vaccinated where rabies has been confirmed previously in humans or dogs. The consumption of dog meat is common in Vietnam, as people believe that eating dog meat improves health and longevity. One study found that two patients who previously had never been bitten by rabid animals were admitted to hospitals, showing clinical signs of rabies [40]. Both patients had been involved in preparing and consuming a dog and a cat, respectively. It was assumed that viral transmission may have occurred during animal slaughtering and preparation, but not by ingestion, as the meat was cooked and shared with other people who did not show any clinical symptoms.

For temporal pattern analysis, the STL and SCS methods have been used in the economic and environmental fields, but have not been utilized commonly in the epidemiology/public health fields [41]. The STL technique is a useful tool for understanding the complexity of time series data while the SCS plot may be helpful to visualize monthly seasonal patterns both between and within groups, during study periods. However, interpretation should be conducted cautiously because the horizontal lines (average for vertical lines) are strongly influenced by large values.

We believe that this study can provide valuable information to better understand the geographical and temporal patterns of rabies PEP as well as developing public health and vaccination policies. In addition, this will help the rabies prevention and control program of Vietnam, where rabies management is taken seriously under a One Health framework established by MOH and MARD (a joint decree to control zoonoses and rabies).

## Conclusions

We found that rabies PEP incidence rates have decreased over time in Vietnam. Overall, there were no significant increase in the number of rabies outbreaks throughout the year. Our findings provide insight into understanding the geographical and seasonal patterns of rabies PEP incidence in Vietnam. This study provides evidence to aid government officials when making policy decisions as well as investing resources. Moreover, these data may be utilized to raise public awareness to prevent exposures, to seek proper medical care and reduce unnecessary PEP.

## Ethics approval and consent to participate

The study was approved by the Hanoi Medical University Institutional Review Board (reference number: 00003121; approval date: 22 Dec 2015), Vietnam.

## Supporting information

S1 Fig This is the rabies data file: Monthly rabies cases at provincial level from 2005 to 2015 in Vietnam. (XLS)
